# Worldwide research trends on tumor burden and immunotherapy: a bibliometric analysis

**DOI:** 10.1097/JS9.0000000000001022

**Published:** 2024-01-04

**Authors:** Lei Zhang, Han Zheng, Shi-Tao Jiang, Yao-Ge Liu, Ting Zhang, Jun-Wei Zhang, Xin Lu, Hai-Tao Zhao, Xin-Ting Sang, Yi-Yao Xu

**Affiliations:** Department of Liver Surgery, Peking Union Medical College Hospital, Chinese Academy of Medical Sciences and Peking Union Medical College (CAMS and PUMC), Beijing, People’s Republic of China

**Keywords:** bibliometric, citespace, hotspot, immunotherapy, tumor burden, VOSviewer

## Abstract

Various immunotherapy has been greatly applied to comprehensive treatment of malignant cancer under different degrees of tumor burden. Scientific researchers have gained considerable progress in the relationship between immunotherapy and tumor burden in recent years. This review aimed to explore the prospect and developing trends in the field of tumor burden and immunotherapy from a bibliometric perspective. Articles about tumor burden and immunotherapy were collected from the Web of Science Core Collection (WoSCC) (retrieved on 3 January 2023). The R package ‘Bibliometrix’ analyzed the primary bibliometric features and created a three-filed plot to display the relationship between institutions, countries, and keywords. VOSviewer was used for co-authorship analysis, co-occurrence analysis, and their visualization. And CiteSpace calculated the citation burst references and keywords. A total of 1030 publications were retrieved from 35 years of scientific researches. The United States (US) and China published the most articles. The most productive journals were *Cancer Immunology Immunotherapy* and *Journal for ImmunoTherapy of Cancer*. The top one institution of the highest output was University of Texas MD Anderson Cancer Center. The hot keywords of strong citation burst strength in recent years were ‘nivolumab’, ‘tumor microenvironment’, and ‘immune checkpoint inhibitor’. The most popular tumor type is melanoma. This bibliometric analysis mapped a basic knowledge structure. The field of tumor burden and immunotherapy is entering a rapid growing stage and keeping it value for future research.

## Introduction

Highlights

- This study is the first bibliometric analysis in the field of tumor burden and immunotherapy.
- The United States occupied an important position in article output and international collaborations.
- Hotspots in future researches involves ‘nivolumab’, ‘tumor microenvironment’, and ‘immune checkpoint inhibitor’.

Immunotherapy has been rapidly matured into a vigorous tool and occupied an important position in cancer therapies especially for unresectable and advanced cancer. No matter stimulating or inhibiting, immunotherapy has multifarious ways to modulate and alter one’s immune system response to treat a disease. Immunotherapy includes various concrete remedies: immune checkpoint inhibitors (ICI)^1–3^, CAR (chimeric antigens receptor) T-cell therapy^4,5^, cancer vaccines^6,7^, cytokines^8,9^, and oncolytic viruses^10^. However, though theoretically promising measure it is, the effect and outcome of immunotherapy to different person is practically individual.

Tumor burden is comprehensively assessed by quantitative or qualitative methods such as imaging (CT scan and FDG-PET), liquid biopsy, or lactate dehydrogenase or serum-based biomarkers (CEA, CA19-9 etc.). High tumor burden has a negative effect on immunotherapy^11^. There are some links between tumor burden and immunotherapy, which may provide a view in the assessment of immunotherapy outcome. Therefore, growing emphasis is put on the research on universal definition of tumor burden, relationship between tumor burden and immunotherapy, and underlying biological mechanisms. However, due to significant heterogeneity of tumor burden and immunotherapy, it is demanding and difficult to conduct clinical studies on a large scale^12^. As more and more scientific researches pay close attention to the field of immunotherapy and tumor burden, a comprehensive analysis and review of such field is expectant.

Bibliometrics is the analysis of publications using statistics to describe or display relationships between published works. Early in 1969, Pritchard put forward bibliometrics, with a definition of ‘the application of mathematical and statistical methods to the computation and analysis of different aspects of textual information to reveal the processes of textual information and the nature and trends in the development of a discipline’^13^. In recent years, bibliometrics is widely used to explored academic publications’ features in a specific research field: influential countries, journals, institutions and authors; favorable publications, references, and keywords^14^. Bibliometrics uses some analyzing tools to generate graphs for visualizing the cooperation among countries, institutions, and authors^15^. Besides, it promotes researchers to quickly grasp the evolution and frontiers of a specific research field.

There have been some bibliometric analyses investigating the pattern and frontiers in the field of immunotherapy^16–18^. Furthermore, bibliometric studies on immunotherapy of various cancers revealed that ‘microsatellite instability’, ‘tumor microenvironment’, ‘tumor mutation burden’, ‘tumor burden’, ‘mutational–landscape’, which related to tumor burden, were currently popular topics in immunotherapy^17,19–21^. These findings indicate that tumor burden and immunotherapy are becoming widely discussed issues. However, bibliometric analysis of this field has not yet been conducted. This review is aimed to fill this gap by creating a general and comprehensive knowledge map of academic publications about tumor burden and immunotherapy.

## Methods

### Data source and search strategy

As one of the most widely accessed academic databases, Web of Science (WoS) accommodates more than 12 000 high-quality journals and comprehensive citation records^22,23^. Therefore, WoS was selected as the target database. In this review, paper search was conducted on 3 January 2023 and relevant literature published since 1987 were exported to the Web of Science Core Collection database (WoSCC). The search strategy was set as follows: [topic search=(tumor burden OR tumor burden OR tumor load OR tumor load) AND (immunotherapy OR immunotherapies OR immunotherapeutic OR immunotherapeutics)]. The type of publication was restricted to article excluding retraction, retracted publication, and book chapter. The language was solely set to English. And related publications were picked up and saved in plain.txt format for further study, complete records and cited references also included^24^.

### Software tools and respective functions

Software tools for bibliometric analysis were Bibliometrix R package^25^, VOSviewer^26^, and CiteSpace^27^. Bibliometrix R package is mainly for quantitative analysis. In Bibliometrix, extraction methods are: authors from the AU field (institutions from AU_UN field and countries from AU_CO field); year of publication from the PY field; keywords from the DE field; citations from the TC field. The functions of Bibliometrix version 4.0.0 in this review were to count the number of publications and their citations, calculate the frequency of used keywords, compute the strength of collaboration among countries/authors, and create a three-field plot of keyword plus analysis.

VOSviewer is a potent tool for co-authorship analysis and co-occurrence analysis. The function of VOSviewer is based on its embedded clustering algorithm^28,29^. In this review, co-authorship analysis was constructed to reveal the teamwork relationships among authors and their institutions, while co-occurrence analysis exhibited the association among different keywords^30,31^. It also advanced the analysis by adding time-overlaying feature to visualize the network over a time span.

CiteSpace is a citation analysis and visualization software. It contributes to visualize the structure, distribution, and trend of academic information, the process of which is also called ‘scientific knowledge mapping’^27^. In this review, CiteSpace was applied to recognize widely cited references/keywords that owned strong citation bursts during a certain period.

**Figure 1:**
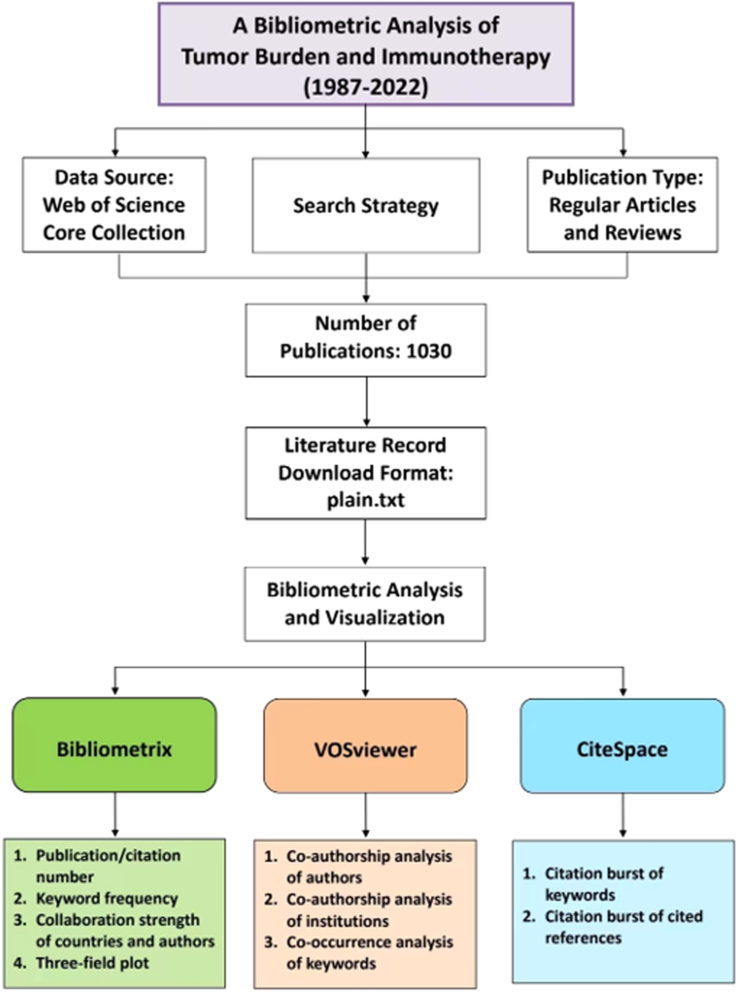
Workflow of the study.

Besides, the online bibliometric website (https://bibliometric.com/) helped to visualize international collaboration. An overview of bibliometric process was shown in Figure 1.

## Results

### Analysis of annual publication output

**Figure 2:**
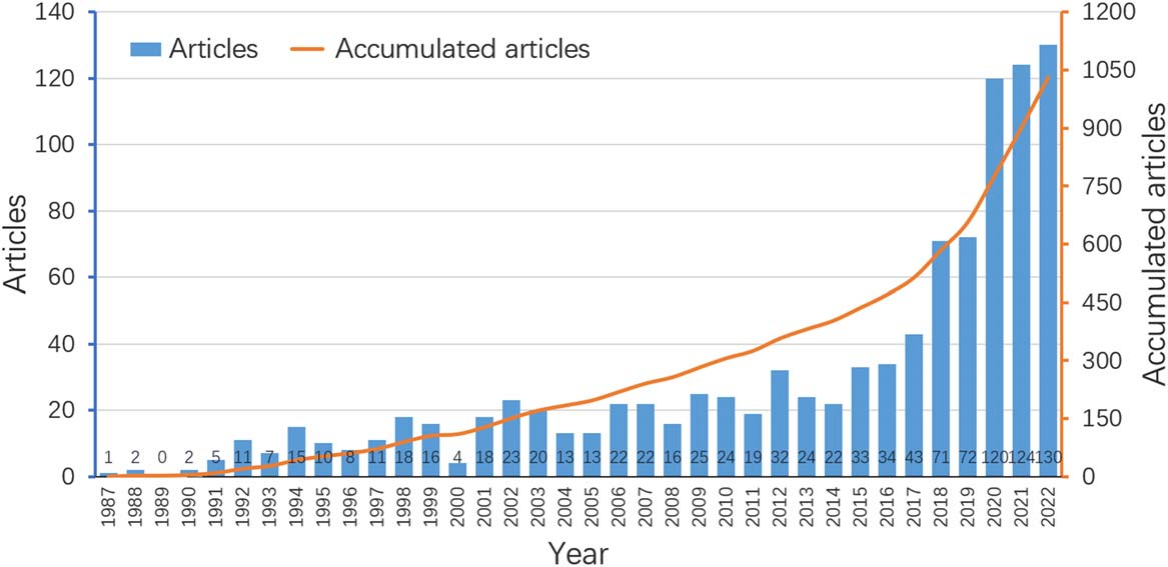
The annual number and the cumulative number of publications.

There were altogether 1030 publications retrieved on the topic of tumor burden and immunotherapy between 1987 and 3 January 2023, spanning 35 years. Figure 2 demonstrates the annual number and the cumulative number of articles related to tumor burden and immunotherapy. A 14.92% annual growth rate was observed. From 1987 to 2017, the cumulative count of publications grew steadily from 1 to 513. In the following 5 years, from 2018 to 2022, the output of publications increased rapidly, getting the accomplishment that the cumulative number of publications reached 1030 in 2022.

### Analysis of national publication volume and collaboration

**Figure 3:**
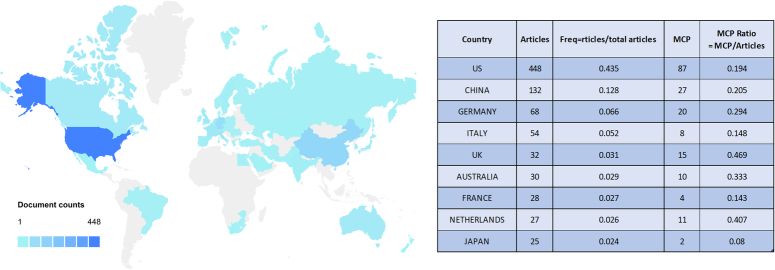
A map of country contribution based on the article output.

After an analysis of national publication output, it was exhibited that a total of 41 countries/regions published articles in relevant field. As shown in Figure 3, the country that contributed the largest volume of publications (*n*=448) was the US, which accounted for 43.5% of the total. China was ranked the second (*n*=132, 12.8%), Germany third (*n*=68, 6.6%), and followed by Italy (*n*=54, 5.2%). Other countries in the top 10 list had less than 40 publications.

Multiple country publications (MCP) revealed the publication number of co-authors from different countries/regions. Although the US had the highest MCP (*n*=87), its MCP ratio (=MCP/articles) was only 19.4%. In Supplementary Figure 1 (Supplemental Digital Content 1, http://links.lww.com/JS9/B635), a further analysis of collaboration among countries/regions was visualized. The most frequent collaboration was from the US to China (frequency=44), and then from the US to Germany (frequency=28), to the United Kingdom (UK) (frequency=21), and to Italy (frequency=15). In the top 10 collaborations, except one collaborative relationship was between Germany and Switzerland, all of these international collaborations were from the US.

### Analysis of institutional output and collaboration

**Figure 4:**
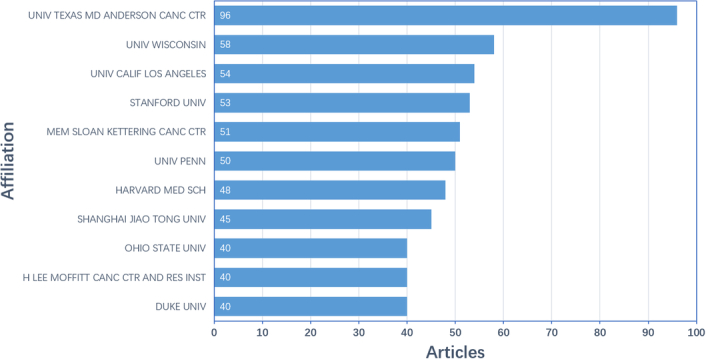
The top 11 institutions with the most publications.

Generally, a total of 1719 institutions conducted researches related to the tumor burden and immunotherapy. The top 11 institutions are listed in Figure 4 (because the Duke University, H. Lee Moffitt Cancer Center and Research Institute, and the Ohio State University published the same number of articles, all of them were included, resulting in 11 institutions on the list). Among these institutions, 10 of 11 were from the US and only one was from China. With the biggest output of 96 publications, the University of Texas MD Anderson Cancer Center ranked No. 1.

Furthermore, co-authorship analysis was carried out to investigate the collaborative relationship among institutions. In the clustering network for the co-authorship analysis (Supplementary Figure 2A, Supplemental Digital Content 1, http://links.lww.com/JS9/B635), the size of the dots represented the number of articles published by relevant institution, the color of the dots showed the institution cluster classified by computer according to the strength of collaboration. In Supplementary Figure 2A (Supplemental Digital Content 1, http://links.lww.com/JS9/B635), 30 institutions were distributed into six clusters. The cluster of red color had the greatest number of institutions, containing seven institutions. In the clustering network of the co-authorship plus time-overlapping analysis (Supplementary Figure 2B, Supplemental Digital Content 1, http://links.lww.com/JS9/B635), the color here reflected the average year of publications for respective institutions in the field of tumor burden and immunotherapy. As shown in Supplementary Figure 2B (Supplemental Digital Content 1, http://links.lww.com/JS9/B635), research institutions represented by University of Michigan and Duke University were early starters in this field. Instead, researchers from Shanghai Jiao Tong University and Sun Yat-Sen University in China were active in recent years in the field of tumor burden and immunotherapy.

### Analysis of article output and impact of journals

There were 360 at all journals taking part in the publication of 1030 articles retrieved in this research. Table 1 listed the top 10 journals and their latest impact factors, sorted by the output of articles. The impact of *Cancer Immunology Immunotherapy* from the US, and *Journal for ImmunoTherapy of Cancer* from the UK were comparable, while the former ranked the first with a total output of 53. In these 10 journals, six were classified in Journal Citation Reports (JCR) Quartile 1 (Q1). Seven publishers are from the US, two from Switzerland, and another publisher from the United Kingdom (UK).

**Table 1:** Top 10 journals with most articles about tumor burden and immunotherapy.

Rank	Journals	Articles	Country	IF	JCR-c
1	Cancer Immunology Immunotherapy	53	US	6.63	Q1
2	Journal for Immunotherapy of Cancer	52	UK	12.469	Q1
3	Cancer Research	34	US	13.312	Q1
4	Cancers	27	Switzerland	6.575	Q1
5	Clinical Cancer Research	25	US	13.801	Q1
6	Journal of Immunology	23	US	5.426	Q2
7	Oncoimmunology	22	US	7.723	Q1
8	Journal of Immunotherapy	20	US	4.912	Q2
9	PLOS ONE	18	US	3.752	Q2
10	Frontiers in Oncology	17	Switzerland	5.738	Q2

### Analysis of author influence and collaboration

A total of 8065 authors participated in the study of tumor burden and immunotherapy. In Table 2, the most productive author was Jeffrey Schlom with eight publications and his H-index was eight. Egesta Lopci was the second influential author (seven publications, H-index=6) and the third was Angelo Castello (six publications, H-index=5).

**Table 2:** Top 10 authors with the most articles about tumor burden and immunotherapy.

Rank	Authors	Articles	H-index	Institution and Country
1	Jeffrey Schlom^46^	8	109	National Cancer Institute, US
2	Egesta Lopci	7	29	Humanitas Clinical and Research Hospital, Italy
3	Angelo Castello	6	13	Humanitas Clinical and Research Hospital, Italy
4	Brian I. Rini	6	121	Cleveland Clinic Taussig Cancer Institute, US
5	Paul M. Sondel	6	75	University of Wisconsin, US
6	Jun Yan	6	65	University of Louisville School of Medicine, US
7	Zvia Agur	5	32	Institute for Medical Biomathematics, Israel
8	Chuanlin Ding	5	24	University of Louisville School of Medicine, US
9	Sofia R. Gameiro	5	6	National Cancer Institute, US
10	Richard Harrop	5	31	Oxford BioMedica, UK

In Supplementary Figure 3A (Supplemental Digital Content 1, http://links.lww.com/JS9/B635), the clustering network of co-authorship analysis among researchers was illustrated, which revealed the collaborative relationships among them. The dot size represented the number of publications by each author, while the color reflected the author clusters of different collaboration strengths. Fifty authors were classified into 11 clusters. These clusters were scattered without forming a large community. There was no collaboration among the different clusters. In Supplementary Figure 3B (Supplemental Digital Content 1, http://links.lww.com/JS9/B635), the time-overlapping map for co-authorship analysis among 30 researchers was visualized. It was observed that researchers Hampartsoum B Barsoumian and Wang Jing were conducting researches actively on tumor burden and immunotherapy newly.

### Research hotspots

#### Most cited publications

The most cited publications in a specific field reveals the research impact. Supplementary Table 1 (Supplemental Digital Content 1, http://links.lww.com/JS9/B635) lists the top 10 most cited publications. As shown in the list, they were published between 1998 and 2017, 60% of which have been cited more than 800 times. The most cited article was ‘Guidelines for the evaluation of immune therapy activity in solid tumors: immune-related response criteria^32^’ published in 2009. The second most cited publication was entitled ‘CD19 CAR-T-cells of defined CD4+:CD8+ composition in adult B-cell ALL patients’, published in the Journal of Clinical Investigation in 2016^33^.

#### Citation burst analysis of references

In Supplementary Figure 4 (Supplemental Digital Content 1, http://links.lww.com/JS9/B635), the top 25 most cited references are presented. The dark blue line represented the citation duration from 1987 to 2022, and the red line showed the burst range of the citation duration. The minimum burst range was 2 years. The most cited reference with the strongest citation burst value was the article entitled ‘Improved survival with ipilimumab in patients with metastatic melanoma’ (citation burst=14.84 from 2012 to 2017), written by Hodi *et al*.^1^. The second most cited reference was the article entitled ‘Cancer regression and autoimmunity in patients after clonal repopulation with antitumor lymphocytes’ (citation burst=7.24), written by Dudley *et al*.^34^. In recent years from 2020 to 2022, citation burst continued for eight articles, of which the highest burst value of 6.59 was from the reference ‘Global cancer statistics 2018: GLOBOCAN estimates of incidence and mortality worldwide for 36 cancers in 185 countries’^35^. The second most popular one of the eight recent burst references was ‘Pembrolizumab plus Chemotherapy in Metastatic Non-Small-Cell Lung Cancer’^36^.

#### Keyword occurrence and co-occurrence analysis

**Figure 5:**
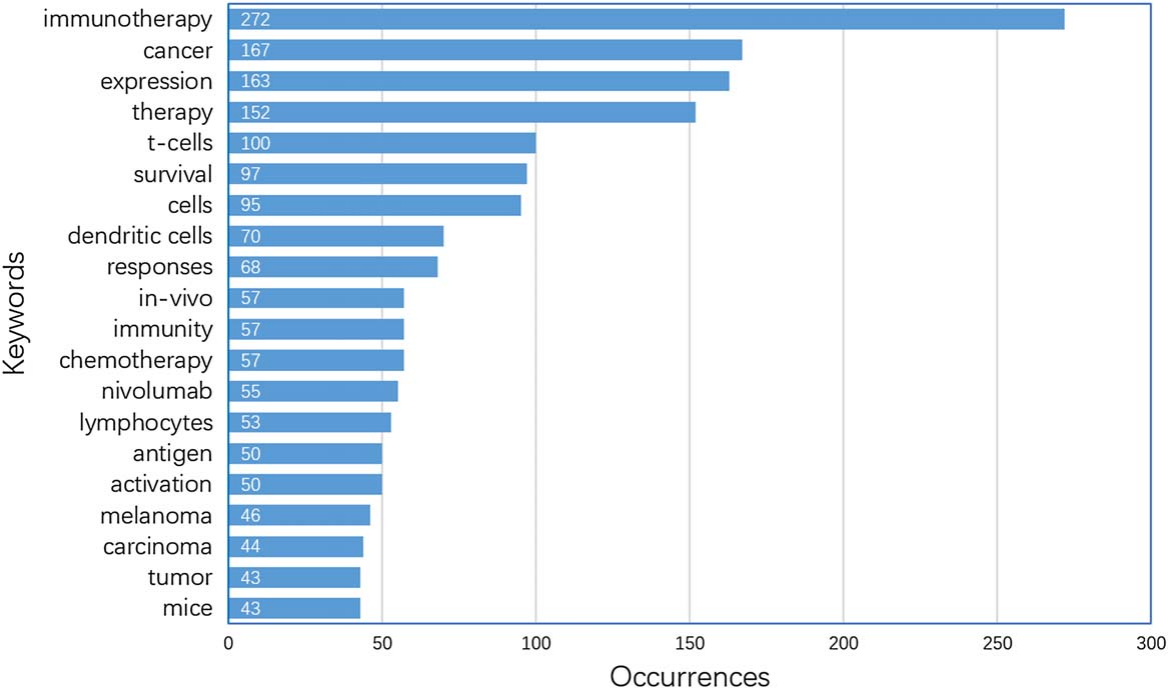
The top 20 most used keywords.

**Figure 6:**
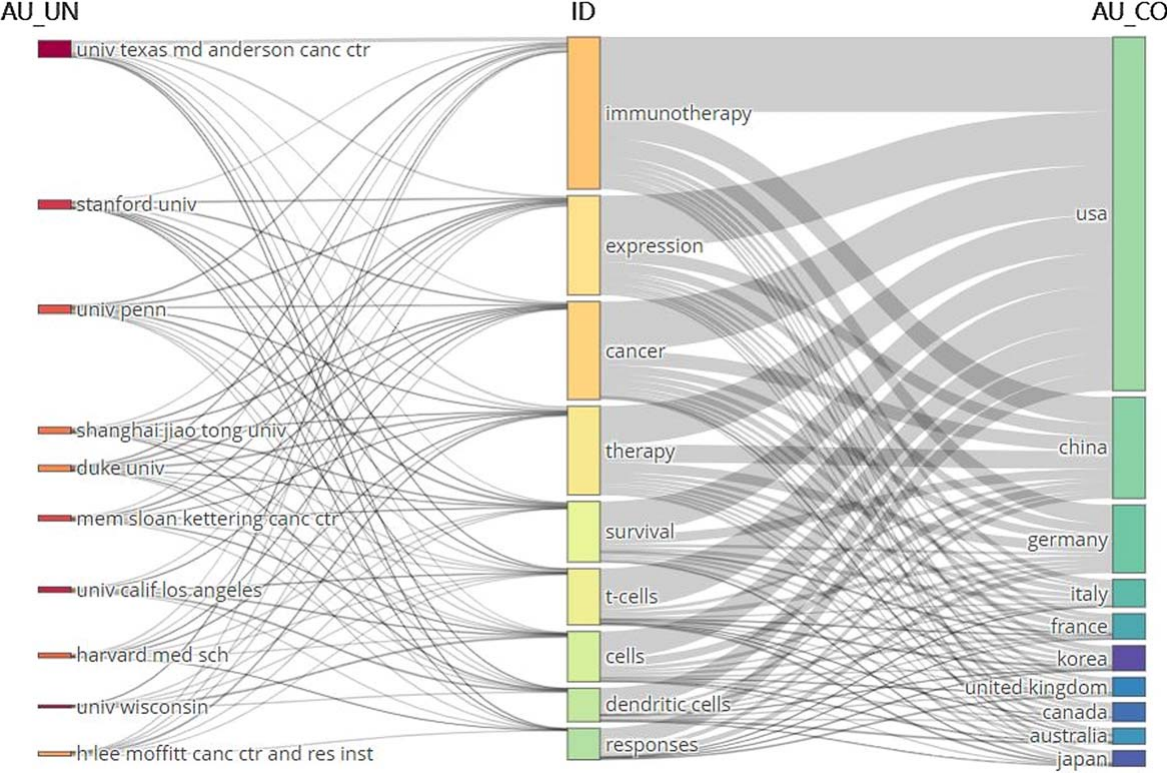
Three-field plot of the keywords plus analysis on tumor burden and immunotherapy (Left field: institutions; Middle field: keywords; Right field: countries).

There were 2722 keywords at all collected in this research. Figure 5 demonstrated the top 20 keywords sorted by occurrence frequency. The keyword ‘immunotherapy’ was used most frequently with 272 occurrences, followed by ‘cancer’ (*n*=167) and ‘expression’ (*n*=163). Among the top 20 keywords, ‘nivolumab’ (*n*=55) was the only monoclonal antibodies and ‘melanoma’ (*n*=46) was the only cancer type that was on the list. Figure 6 further displayed the proportion of core topics for each institution and country, demonstrating the association and distribution among countries, institutions, and keywords in the field of tumor burden and immunotherapy. Generally, almost all the institutions and countries contributed to the nine topics represented by the keywords. However, differences existed. In the aspect of institutions, University of California-Los Angeles were more interested in ‘cancer’, ‘t-cells’, ‘cells’, and ‘dendritic cells’, while University Wisconsin took more part in ‘immunotherapy’ and ‘cells’, and H. Lee Moffitt Cancer Center and Research Institute focused more on ‘therapy’. In the aspect of countries, the US and China contributed largely to all these hotspots. And among these nine keywords of ‘immunotherapy, expression, cancer, therapy, survival, t-cells, cells, dendritic cells, responses’, Canada was less interested in ‘survival, responses’, while Australia and Japan less focused on ‘responses’.

**Figure 7:**
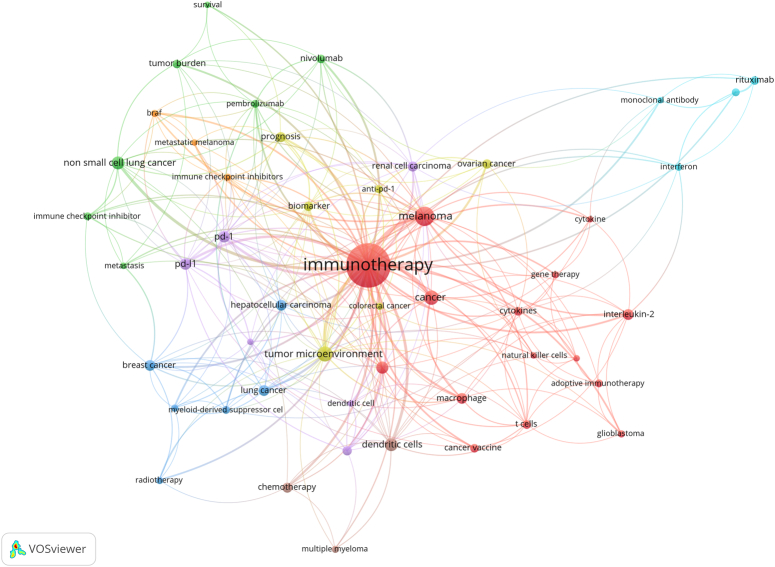
Keyword co-occurrence network.

**Figure 8:**
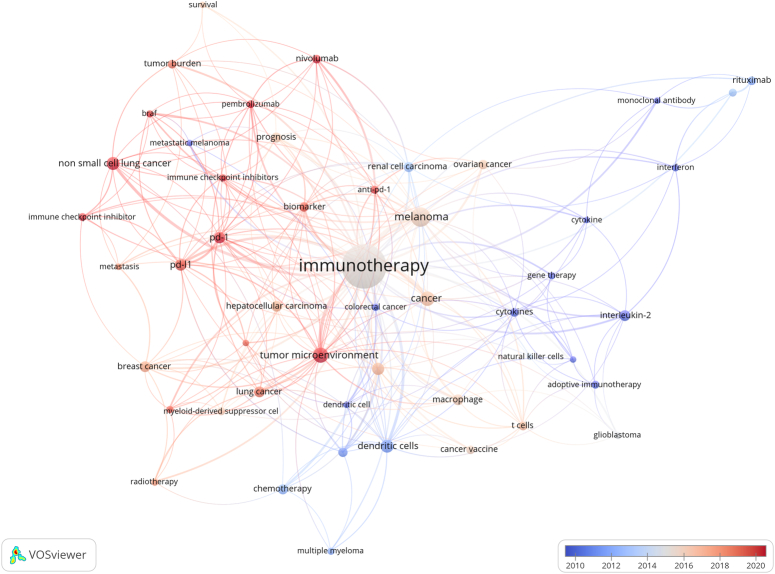
Keyword co-occurrence plus time-overlapping network.

Fifty keywords were included in the co-occurrence analysis. The network of these keywords is displayed in Figure 7. The size of dots represented use-frequency of keyword, the color reflected the keyword cluster, and the distance between dots indicates the intensity of their relationship. Keywords that were more closely correlated were classified into the same cluster. The 50 keywords were divided into eight clusters. Cluster 1 was red (contained 15 keywords), in which primary keywords focused on some compositions of immune system, such as ‘cytokines’, ‘interleukin-2’, ‘macrophage’, ‘t-cells’, ‘natural killer cells’, some kinds of immunotherapy such as ‘cancer vaccine’, ‘adoptive immunotherapy’, ‘gene therapy’, and ‘cancer’ types like ‘melanoma’, ‘glioblastoma’. Cluster 2 was green (seven keywords) with focus on broadly used ‘immune checkpoint inhibitor’ like ‘nivolumab’ and ‘pembrolizumab’. Besides, some keywords such as ‘tumor burden’, ‘metastasis’, and ‘survival’ were contained in Cluster 2. Cluster 3 was blue (six keywords) and focused attention on the various tumor types such as ‘lung cancer’, ‘hepatocellular carcinoma’, and ‘breast cancer’, while keywords like ‘myeloid-derived suppressor cells’ and ‘radiotherapy’ also contained in this cluster. Cluster 4 in yellow (six keywords) included some interesting keywords such as ‘tumor microenvironment’, ‘anti-pd-1’, ‘biomarkers’, and ‘prognosis’ except of two tumor types ‘ovarian cancer’ and ‘colorectal cancer’. Cluster 5 in purple mainly included keywords ‘PD-1’, ‘PD-L1’, and another cancer type ‘renal cell carcinoma’. The Cluster 6 colored in light blue included mainly ‘rituximab’, ‘monoclonal antibody’, and ‘interferon’. The remaining two clusters in orange and brown contained some other keywords, for example, ‘BRAF’, ‘metastatic melanoma’, ‘multiple myeloma’, and ‘chemotherapy’. Figure 8 shows the time-overlapping analysis network of these co-occurrence keywords. Color from dark blue to light blue and from light red to dark red, represents the average active year of these keywords attracting researchers. Early periods of research focused primarily on ‘cytokine/cytokines’, ‘interferon’, and ‘monoclonal antibodies’. On the contrary, topics of ‘tumor microenvironment’, ‘biomarker’, ‘nivolumab’, ‘non-small-cell lung cancer’, and ‘immune checkpoint inhibitor/inhibitors’ occupied a more primary position in recent years.

#### Citation burst analysis of keywords

**Figure 9:**
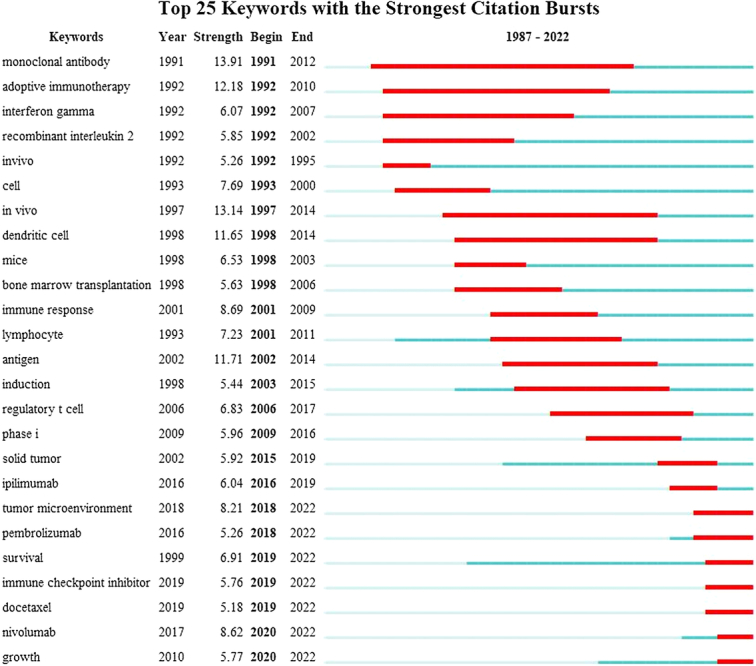
The top 25 keywords with robust citation bursts.

In Figure 9, it presents the top 25 keywords with the strongest citation bursts. The keywords ‘monoclonal antibody’ (burst duration from 1991 to 2012, 21 years) and ‘adoptive immunotherapy’ (1992–2010, 18 years) experienced the most persistent attention over time. In addition, keywords such as ‘tumor microenvironment’ (burst duration from 2018 to 2022), ‘pembrolizumab’ (2018–2022), ‘survival’ (2019–2022), ‘immune checkpoint inhibitor’ (2019–2022), ‘docetaxel’ (2019–2022), ‘nivolumab’ (2020–2022), and ‘growth’ (2020–2022) were attractive more recently, revealing that these keywords represented the popular research topics in recent years and even in the near future.

#### Analysis of different tumor types

In Table 3, it clearly displays the most popular tumor types in the topic of tumor burden and immunotherapy. We counted the total article numbers of different tumor types. Cancer that possessed less than 10 papers like mesothelioma, multiple myeloma, bone cancer, cervical cancer and et.al were classified into the ‘other’ type. Publications that covered more than one tumor types were also included in the ‘other’ type. Supplementary Table 2 (Supplemental Digital Content 1, http://links.lww.com/JS9/B635) shows the top 5 articles ranked by their total citation of the top 5 tumor types. Melanoma ranks the first place, owning as much as 138 publications. The second one is leukemias and lymphoma, which represent the majority of hematologic malignancies. Lung cancer, breast cancer, and ovarian cancer are ranked third to fifth, respectively.

**Table 3:** Top 13 interesting tumor types.

Tumor type	Number
Melanoma	138
Leukemias and lymphoma	122
Lung cancer	93
Breast cancer	65
Ovarian cancer	45
Renal cancer	44
Brain cancer	43
Liver cancer	38
Colorectal cancer	34
Prostate cancer	23
Pancreatic cancer	17
Bladder cancer	12
Head and neck cancer	10
Other	346
Total	1030

## Discussion

This review analyzed the publications in the field of tumor burden and immunotherapy from 1987 to 2022 through a bibliometrics analytical method. The first article was published in 1987. In this article of murine models, Ottow *et al*.^37^ firstly found that immunotherapy with interleukin-2 and lymphokine-activated killer cells may reduce tumor burden for intraperitoneal cancer. Publication growing trend could be divided into one slow and one rapid phase according to the number of annual publications. The slow growth phase was from 1987 to 2017 with less than 50 publications per year. 2018 witnessed a great progress of annual publication output in the study of tumor burden and immunotherapy. From 2018 to 2022, relevant studies were in a rapid growth phase, with the annual number of publications exceedingly over 70 each year. It indicates that the field of tumor burden and immunotherapy may still be in an active and robust stage in the following years. The potential reason hidden behind this phenomenon might be that personalized medicine more and more enters people’s sight^38^. As an individualized index, tumor burden would influence the effect and results of immunotherapy of different individuals. In order to provide more accurate and effective immunotherapy for patients, more institutions have been putting more attention and support on this field, contributing to the high growth rate in recent years.

In this research field, the top 10 countries accounted for 84.0% of the total publications. The US dominated in these field with the greatest output of publications and also occupied an important position of international collaborations. As exhibited above, in the field of tumor burden and immunotherapy, the US takes the leadership position and stands for the global frontier level. Despite its powerful economy, the great investment in health care also contributes its accomplishment. National economy support and international collaborations will further promote the overall development of this field.

Since the US had the largest number of total publications, the result that almost all the top 11 institutions were from the US was reasonable. China, though ranked the second concerning the publication number, had only one institution in the top 11 list. Ranked third in publication output, Germany; however, had no institution in the top 11. As for the recent activity degree, two institutions in China, Shanghai Jiao Tong University and Sun Yat-Sen University, performed most vigorously in these years. Based on international teamwork, research competitiveness would be improved, suggesting that it is of great importance to seek extensive collaboration among institutions especially when economic or resources are limited.

As for journal impact, the impact factor^39^ and JCR^40^ were potent indicators to value the journals’ impact. Among top 10 journals, JCR Q1 journals account for 60%, while only two of top 10 journals have publications more than 50 in research of tumor burden and immunotherapy. Among them, journals named *Cancer Immunology Immunotherapy* and *Journal for ImmunoTherapy of Cancer* has the greatest number of publications. Core journals often take up the responsibility of publishing essential studies in relevant field. Thus, these top journals could be recommended for researchers to submit their work. Moreover, although China and Japan contributed significantly to this field, no Asian publisher take charge of the top 10 journals. It indicates that China and Japan have enough capability to establish a journal with international influence.

One of the problems this study hoped to resolve was what the scientific hotspots were that researchers widely followed in the field of tumor burden and immunotherapy. Research hotspots could be analyzed from various aspects: publications, references, and keywords.

The citation number of a publication could be one of the indicators of its impact^41^. Frequently cited publications reflected core topics in a specific research field, which helped to identify research hotspots. Generally, these top 10 cited publications focused on the following topics: the response criteria of immunotherapy, novel strategies of immunotherapy, safety of immunotherapy, and management negative effect of immunotherapy.

Early in 1998, Khouri *et al*.^42^ from the University of Texas M.D. Anderson Cancer Center explored a remedy of fludarabine-nonablative chemotherapy plus allogeneic hematopoietic transplantation for lymphoid malignancies. This scenario was found feasible; however, more promising in patients with lower tumor burden. Then in 2006, Kershaw *et al*.^43^ utilized a novel adoptive immunotherapy method with gene-redirected t-cells to treat metastatic ovarian cancer. Although patients receiving such approach experienced mild side effects with grade 1 to 2 treatment-related toxicity, no reduction in tumor burden was detectable in all patients. In the study of Carpenito *et al*.^44^, chimeric antimesothelin t-cells were created and transferred intratumorally or intravenously into mice engrafted with large pre-established tumors. The results showed that the engineered t-cells reduced the tumor burden, and even resulted in complete eradication of the tumors in some cases, although it was just in animal experimental stage. Late in 2011, Brentjens *et al*.^45^ conducted an adoptive immunotherapy using CD19-targeted t-cells to treat refractory B-cell leukemias. The results showed that approach is promising and more likely to be beneficial in patients with low tumor burden. These four articles described different adoptive immunotherapy methods with different impact on tumor burden. Furthermore, Turtle *et al*.^33^, in a study of CD19-specific CAR-T-cell manufactured from defined CD4+ and CD8+ t-cell composition to treat B-cell acute lymphoblastic leukemia (ALL), deeply revealed that high tumor burden might increase the risks of severe cytokine release syndrome and neurotoxicity. In addition, researchers also identified some serum biomarkers that indicated patients who might be at the highest risk of toxicity, such as serum ferritin, C-reactive protein, IL-6, and IFN-γ.

In the most cited publication, Wolchok *et al*.^32^ contributed to build a systemic criterion for immune-related response observed in immunotherapy. In this phase II clinical trial program with ipilimumab, researchers observed that ipilimumab still responded even when tumor burden increased initially, resulted in a favorable survival. Similar to this study, Weber *et al*.^46^ also explored responses of ipilimumab for cancer and its immune-related adverse events (irAEs). The similar response pattern that ipilimumab resulted regression after initial increase of tumor burden was also seen in this publication. Interestingly, the researchers revealed that irAEs correlated with treatment response in some studies.

Immunotherapy, radiotherapy, and chemotherapy are three major pillars for cancer management. In the study of Lee *et al*.^47^, researchers reported that the treatment effect of tumor burden reduction after ablative radiotherapy depended largely on CD8(+) t-cell responses, which could be greatly amplified by local immunotherapy. It indicated that immunotherapy could not only decrease tumor burden by itself but also via a radiotherapy way. However, chemotherapy as another way for killing tumor cells, may impede the path of immunotherapy. In 2010, a paper published by Park *et al*.^48^ showed that the addition of chemotherapeutic drugs, although helped to reduce tumor burden, could abrogate the effect of immunotherapy, leading to an unstable status when rechallenge or earlier relapse happened. The influence toward tumor burden among immunotherapy, chemotherapy, and radiotherapy needs further exploration.

Although immunotherapy showed its promoting prospect, there was still a subgroup of patients who appeared as ‘hyperprogressors’ with accelerated tumor growth and clinical deterioration compared with pretherapy. In the study of Kato *et al*.^49^, an analysis of genomic alterations was conducted to evaluate and explore the accurate changes related with accelerated tumor advance. The analysis results were MDM2 family amplification or EGFR aberrations, which could become a new index of tumor burden to help identify patients at risk of hyperprogression on immunotherapy.

Keywords stand for the core content of the research, while keyword frequency show the keyword impact in special field. These keywords represent another aspect of research hotspots. The most frequently used keywords were mainly related to the composition of ‘immunity’, such as ‘antigen’, ‘expression’, ‘activation’, ‘t-cells’, ‘cells’, ‘dendritic cells’, and ‘lymphocytes’. A group of similar meaning of keywords included ‘cancer’, ‘tumor’, and ‘carcinoma’, with an only concrete type of ‘melanoma’. Besides, ‘nivolumab’ was the only ICI drug on the list. Time-overlapping analysis; however, is used not to identify the most frequently used keywords, but the most recently used keywords. The results of ‘tumor microenvironment’, ‘biomarker’, ‘nivolumab’, ‘non-small-cell lung cancer’, and ‘immune checkpoint inhibitor/inhibitors’ represented the recent research hotspots.

Citation burst analysis is a method provided by CiteSpace to primarily provide reference and keywords that have significant shifts in a specific period. In the citation burst analysis of references/keywords, citation strength is an index to reveal the attraction intensity of a reference/keyword that is widely followed and discussed. And the time of citation burst represents the duration of its citation and whether it gets the latest attentions in this field. It also provides an aspect of research hotspots. In this study, ‘nivolumab’, ‘tumor microenvironment’, ‘immune checkpoint inhibitor’ were the keywords with a latest burst until 2022. All of them also appeared before in the time-overlapping analysis. As for references, there were eight cited publications since 2020 and the burst has persisted until 2022. Four of them focused on different kinds of ICIs like pembrolizumab and atezolizumab with or without chemotherapy for treatment of various cancer^2,36,50,51^. One of them explored the first FDA approved CAR-T-cell therapy called tisagenlecleucel, to treat refractory B-cell acute lymphoblastic leukemia^52^. One evaluated the incidence and mortality of various cancer worldwide^35^and one established guideline for immunotherapy responses^53^. Besides, one reference focused on relevant circulating T-cell subgroup as a predictive factor for response to PD-1 inhibitor^54^. Reference hotspots were consistent with keyword hotspots, that was, various indicators of tumor burden were used to predict the response and outcome of various immunotherapy like CAR-T-cell adoptive immunotherapy and ICI immunotherapy. In the near future, research directions of interest may lie in these topics: 1. Make sure the superficial impact of tumor burden on various immunotherapy response including T-cell adoption and ICIs; 2. Excavate indicators of tumor burden with clinically practical value; 3. Explore the deep mechanisms underlining the relationship between tumor burden and immunotherapy.

As for different tumor types, melanoma, leukemias and lymphoma, lung cancer, breast cancer are the top 1–4 interesting hotspots, each having more than 50 publications in the field of immunotherapy and tumor burden. ICI was first successfully applied and approved in patients with advanced melanoma. Therefore, it is reasonable that this tumor type of melanoma contains the most publications on this topic.

After reviewing these publications, the following clues could be concluded about the relationships between tumor burden and immunotherapy. First of all, tumor burden is an important index of evaluation for judging the therapeutic effect of immunotherapy. Then, there are more and more peripheral blood molecules such as ctDNA and circulating tumor cells, to indirectly monitoring the tumor burden and furtherly monitoring the immunotherapy effect^33,55–58^. Secondly, immunotherapy response has four patterns: (a) tumor burden decreased without new lesions; (b) stable tumor burden; (c) response after tumor burden increased; (d) tumor burden rapidly increased and cancer hyperprogression^32,46^. Moreover, for the most part, lower tumor burden before immunotherapy is relative to better immunotherapeutic outcome^45,59–61^. And higher tumor burden is related to more adverse events of immunotherapy^46^. Accumulating evidence suggests that regulatory T-cells (Tregs) and myeloid-derived suppressor cells (MDSC) are elevated as tumor burden increased^62^, and Tregs and MDSC are related to tumor immune tolerance^63^. Thus, it is understandable that lower tumor burden is possibly related to lower tumor tolerance and better outcome of immunotherapy.

It must be acknowledged that this review owns several modest limitations. First of all, as a bibliometric analysis, data collection and procession highly depend on software. Although such analysis cannot wholly substitute for system retrieval, it facilitates a comprehensive analysis from great data. Secondly, only articles written in English from the WoSCC database were collected in this study, that means some valuable studies may be missed. Since WoSCC has a high coverage rate of the large majority of studies, it is considered that such overlook would not significantly influence the general trends. Thirdly, since delay exists in citation impact, some high-quality studies recently published may be underestimated on their impact, which need to be followed and updated in future studies. Despite all these, this study will academically help researchers to understand the developing trend, hotspots and frontiers in the field of tumor burden and immunotherapy.

## Conclusions

In conclusion, relationship between tumor burden and immunotherapy has grasped increasing attention and relevant researches are in a highly developing stage. New clues of great concern for future research hotspots are: 1. impact of tumor burden on various immunotherapy response; 2. indicators of tumor burden with clinically practical value; 3. mechanisms behind the relationship between tumor burden and immunotherapy. With such analysis results, researchers are well armed for a more accurate and deeper study in the field of tumor burden and immunotherapy.

## Ethical approval

No ethical approval and patient consent were required for all analyses were based on literature research.

## Consent

Not required.

## Sources of funding

This article was partially sponsored by the National High Level Hospital Clinical Research Funding (2022-PUMCH-A-237) and the National High Level Hospital Clinical Research Funding (2022-PUMCH-C-049).

## Author contribution

L.Z. and H.Z.: wrote the manuscript and data interpretation; S.-T.J.: performed the data analysis and modified the manuscript; Y.-G.L., T.Z., and J.-W.Z.: participated in the design of the study; X.L., H.-T.Z., X.-T.S., and Y.-Y.X.: conceived and designed the study. All authors contributed to the article and approved the submitted version.

## Conflicts of interest disclosure

The authors declare that the research was conducted without any commercial or financial relationships that could be construed as a potential conflict of interest.

## Research registration unique identifying number (UIN)

1. Name of the registry: not applicable.
2. Unique identifying number or registration ID: not applicable.
3. Hyperlink to your specific registration (must be publicly accessible and will be checked): not applicable.

## Guarantor

Yiyao Xu, Department of Liver Surgery, Peking Union Medical College (PUMC) Hospital, PUMC and Chinese Academy of Medical Sciences, Dongcheng, Beijing 100730, People’s Republic of China. E-mail: xuyiyao@pumch.cn.

Lei Zhang, Department of Liver Surgery, Peking Union Medical College (PUMC) Hospital, PUMC and Chinese Academy of Medical Sciences, Dongcheng, Beijing 100730, People’s Republic of China. E-mail: zhanglei_tj@163.com.

## Provenance and peer review

Not commissioned, externally peer-reviewed.

## Data availability statement

The data in this review is not sensitive in nature and is accessible in the public domain. The data is therefore available and not of a confidential nature. Additional data are made available in supplementary material of this manuscript.

## Supplementary Material

SUPPLEMENTARY MATERIAL
