# Supplementary material for: Worldwide research trends on tumor burden and immunotherapy: a bibliometric analysis
Source: Int J Surg. 2024 Jan 4;110(3):1699–710. doi: 10.1097/JS9.0000000000001022 (PMC10942200; doi:10.1097/JS9.0000000000001022)
Supplement: SUPPLEMENTARY MATERIAL [file js9-110-1699-s001.docx]

Supplementary Material

Worldwide Research Trends on Tumor Burden and Immunotherapy: A Bibliometric Analysis

Lei Zhang, Han Zheng, Shi-Tao Jiang, Yao-Ge Liu, Ting Zhang, Jun-Wei Zhang, Xin Lu, Hai-Tao Zhao, Xin-Ting Sang, Yi-Yao Xu*

*** Correspondence:** Yi-Yao Xu: xuyiyao@pumch.cn

# Supplementary Figures and Tables

## Supplementary Figures


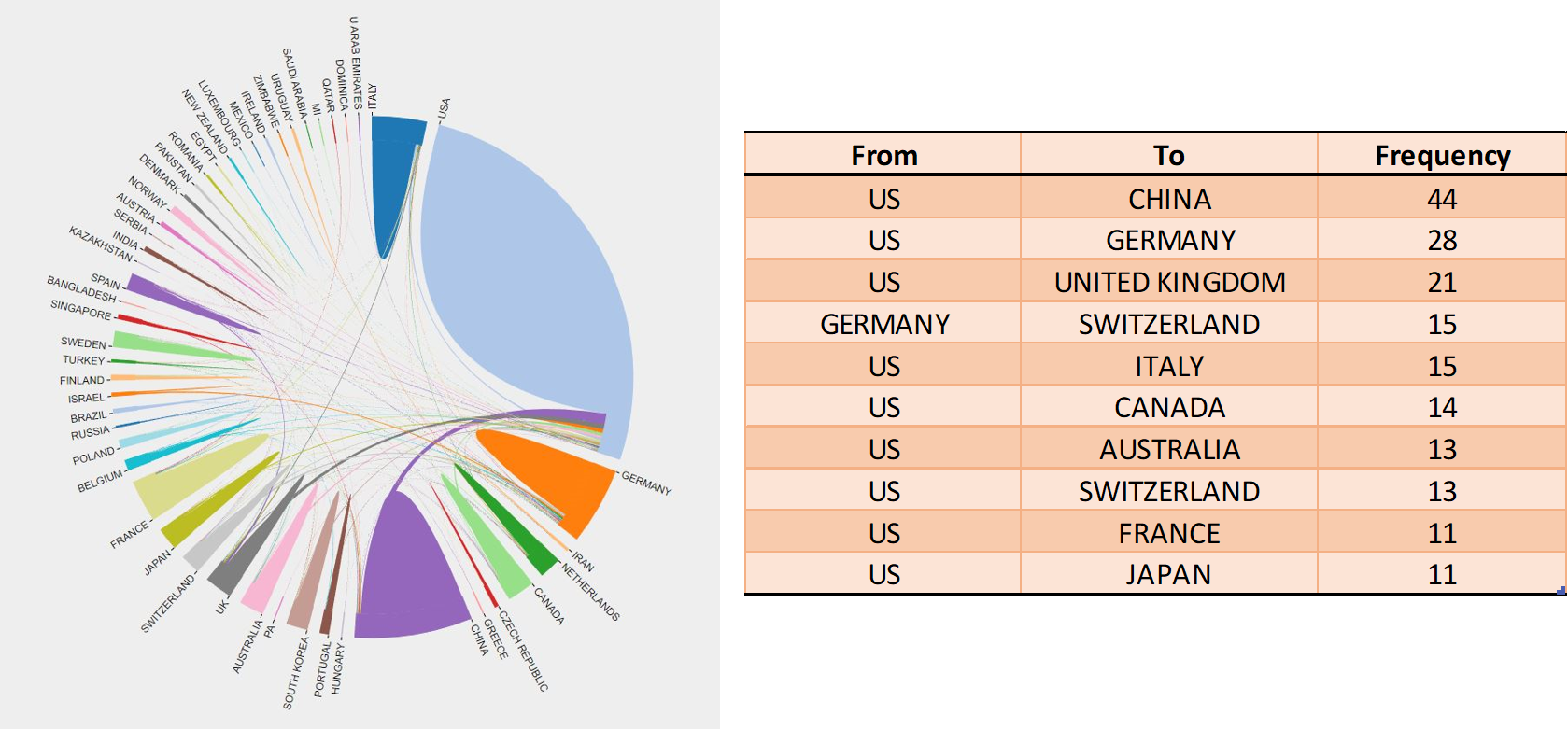


**Supplementary Figure 1.** International collaboration and high frequency collaboration countries in the field of tumor burden and immunotherapy.


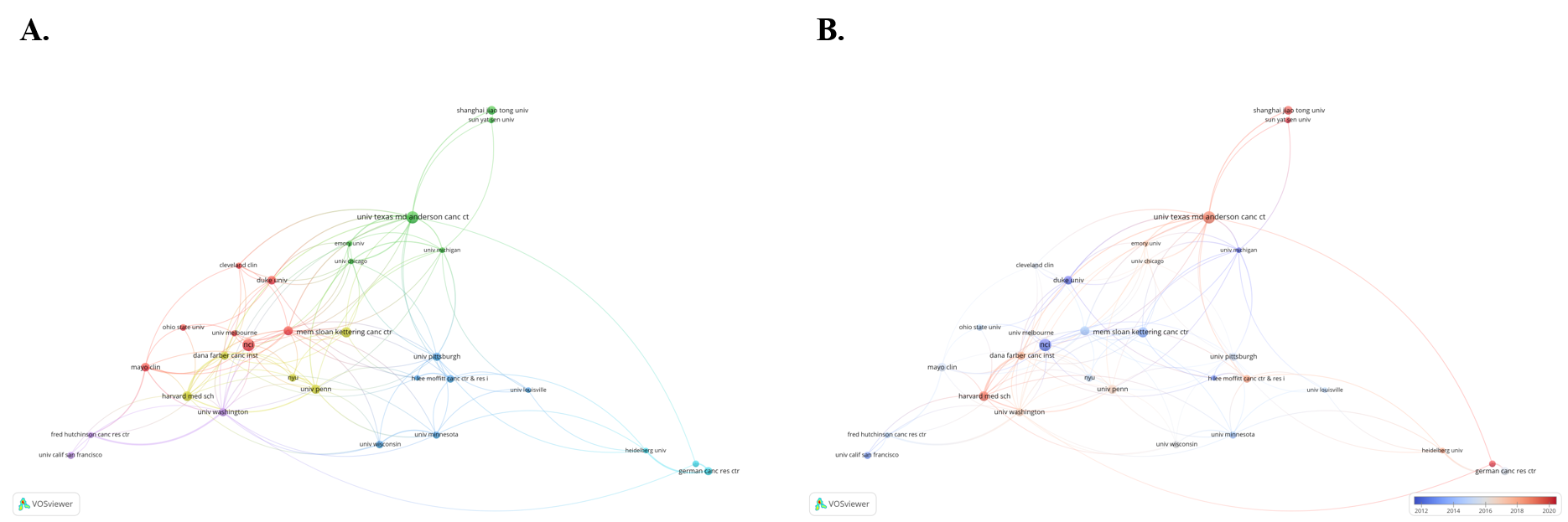


**Supplementary Figure 2.** Clustering network and time-overlapping visualization for institutional co-authorship analysis


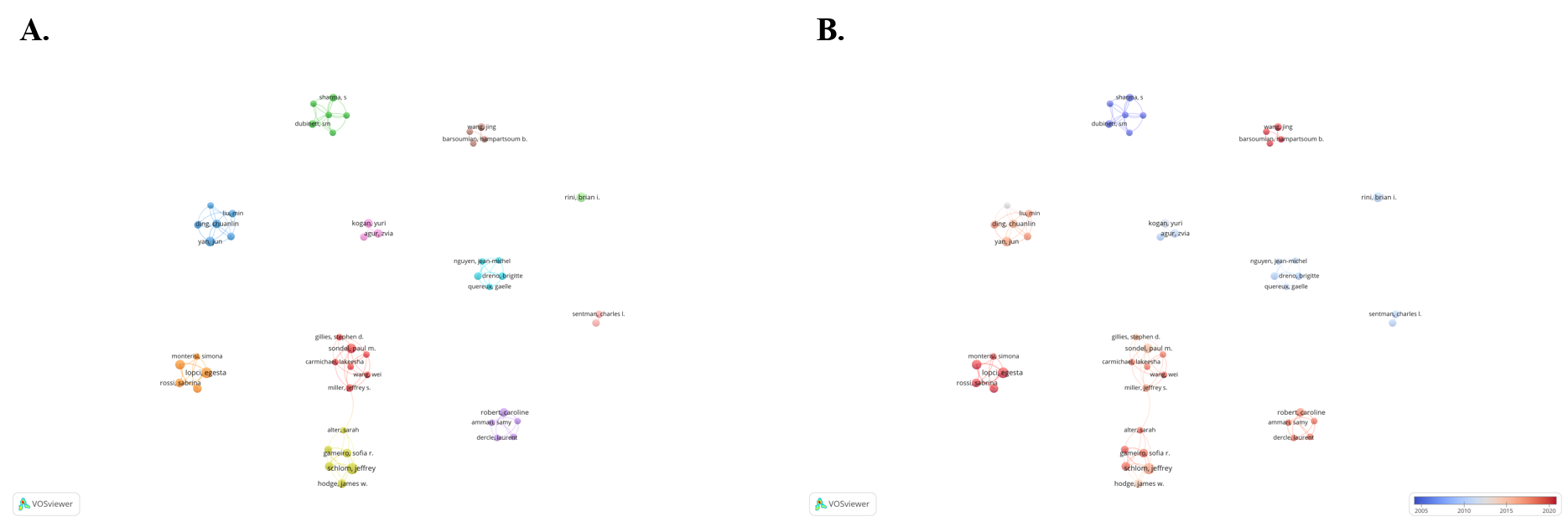


**Supplementary Figure 3.** Clustering network and time-overlapping visualization for author co-authorship analysis


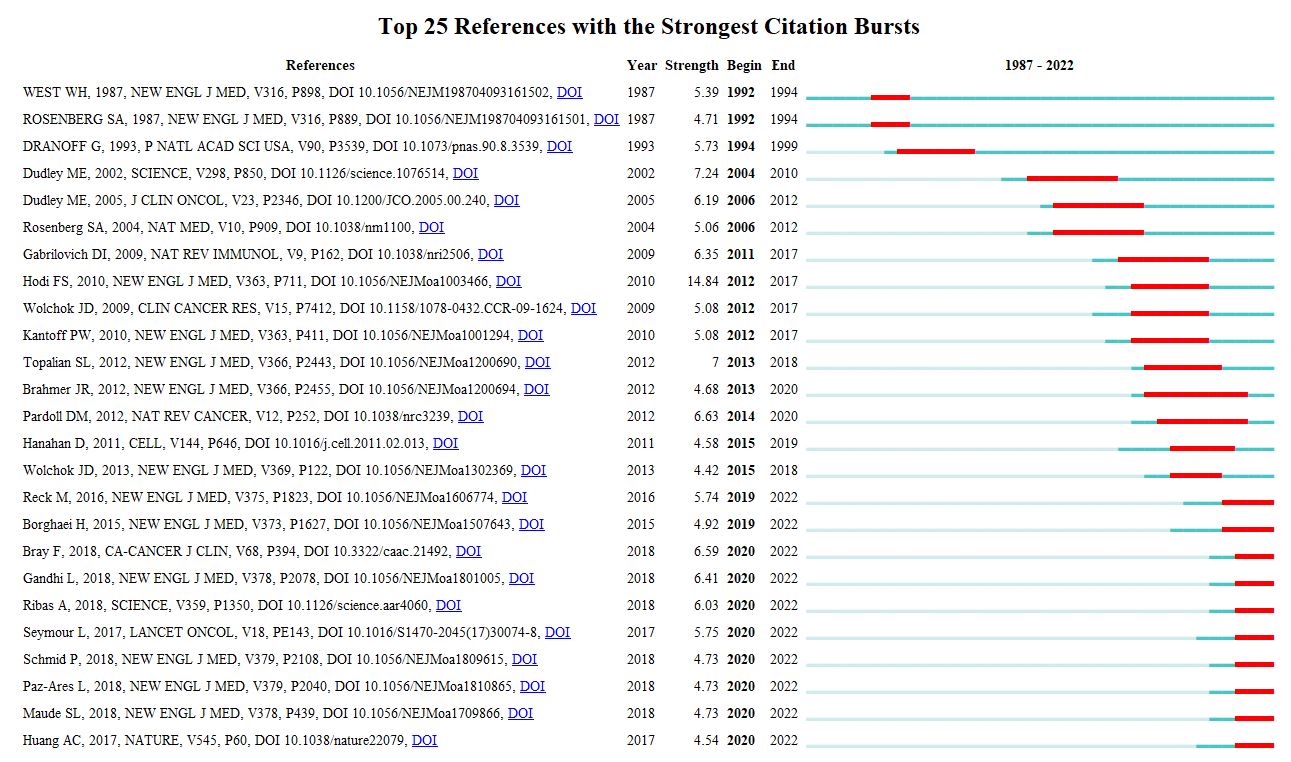


**Supplementary Figure 4.** Top 25 cited references with the strongest citation bursts on tumor burden and immunotherapy

## Supplementary Tables

**Supplementary Table 1**. The top 10 cited publications

| **Rank** | **Title** | **Year, Journal** | **First author** | **Total Citations** | **TC per Year** |
| --- | --- | --- | --- | --- | --- |
| 1 | Guidelines for the evaluation of immune therapy activity in solid tumors: immune-related response criteria | 2009, CLIN CANCER RES | WOLCHOK JD | 2299 | 153.27 |
| 2 | CD19 CAR-T cells of defined CD4+:CD8+ composition in adult B cell ALL patients | 2016, J CLIN INVEST | TURTLE CJ | 1168 | 146.00 |
| 3 | Management of immune-related adverse events and kinetics of response with ipilimumab | 2012, J CLIN ONCOL | WEBER JS | 1002 | 83.50 |
| 4 | Safety and persistence of adoptively transferred autologous CD19-targeted T cells in patients with relapsed or chemotherapy refractory B-cell leukemias | 2011, BLOOD | BRENTJENS RJ | 917 | 70.54 |
| 5 | Therapeutic effects of ablative radiation on local tumor require CD8+ T cells: changing strategies for cancer treatment | 2009, BLOOD | LEE Y | 913 | 60.87 |
| 6 | A phase I study on adoptive immunotherapy using gene-modified T cells for ovarian cancer | 2006, CLIN CANCER RES | KERSHAW MH | 853 | 47.39 |
| 7 | Transplant-lite: induction of graft-versus-malignancy using fludarabine-based nonablative chemotherapy and allogeneic blood progenitor-cell transplantation as treatment for lymphoid malignancies | 1998, J CLIN ONCOL | KHOURI IF | 736 | 28.31 |
| 8 | Control of large, established tumor xenografts with genetically retargeted human T cells containing CD28 and CD137 domains | 2009, PROC NATL ACAD SCI U S A | CARPENITO C | 622 | 41.47 |
| 9 | Hyperprogressors after Immunotherapy: Analysis of Genomic Alterations Associated with Accelerated Growth Rate | 2017, CLIN CANCER RES | KATO S | 544 | 77.71 |
| 10 | The therapeutic effect of anti-HER2/neu antibody depends on both innate and adaptive immunity | 2010, CANCER CELL | PARK SG | 375 | 26.79 |

**Supplementary Table 2**. The top 5 cited publications of 5 tumor types

| **Tumor Type** | **Paper** | **Title** | **Total Citations** | **TC per Year** |
| --- | --- | --- | --- | --- |
| Melanoma | WOLCHOK JD, 2009, CLIN CANCER RES | Guidelines for the evaluation of immune therapy activity in solid tumors: immune-related response criteria | 2299 | 153.27 |
|  | WEBER JS, 2012, J CLIN ONCOL | Management of immune-related adverse events and kinetics of response with ipilimumab | 1002 | 83.50 |
|  | LEE Y, 2009, BLOOD | Therapeutic effects of ablative radiation on local tumor require CD8+ T cells: changing strategies for cancer treatment | 913 | 60.87 |
|  | STOKLASEK TA, 2006, J IMMUNOL | Combined IL-15/IL-15Ralpha immunotherapy maximizes IL-15 activity in vivo | 282 | 15.67 |
|  | ENGELAND CE, 2014, MOL THER | CTLA-4 and PD-L1 checkpoint blockade enhances oncolytic measles virus therapy | 202 | 20.20 |
| Leukemias and Lymphoma | TURTLE CJ, 2016, J CLIN INVEST | CD19 CAR-T cells of defined CD4+:CD8+ composition in adult B cell ALL patients | 1168 | 146.00 |
|  | BRENTJENS RJ, 2011, BLOOD | Safety and persistence of adoptively transferred autologous CD19-targeted T cells in patients with relapsed or chemotherapy refractory B-cell leukemias | 917 | 70.54 |
|  | KHOURI IF, 1998, J CLIN ONCOL | Transplant-lite: induction of graft-versus-malignancy using fludarabine-based nonablative chemotherapy and allogeneic blood progenitor-cell transplantation as treatment for lymphoid malignancies | 736 | 28.31 |
|  | SCHMID C, 2007, J CLIN ONCOL | Donor lymphocyte infusion in the treatment of first hematological relapse after allogeneic stem-cell transplantation in adults with acute myeloid leukemia: a retrospective risk factors analysis and comparison with other strategies by the EBMT Acute Leukemia Working Party | 355 | 20.88 |
|  | SATO K, 2003, IMMUNITY | Regulatory dendritic cells protect mice from murine acute graft-versus-host disease and leukemia relapse | 265 | 12.62 |
| Lung Cancer | MAIER B, 2020, NATURE | A conserved dendritic-cell regulatory program limits antitumour immunity | 198 | 49.50 |
|  | WANG M, 1995, J IMMUNOL | Active immunotherapy of cancer with a nonreplicating recombinant fowlpox virus encoding a model tumor-associated antigen | 187 | 6.45 |
|  | GRIFFITH SD, 2019, ADV THER | Generating Real-World Tumor Burden Endpoints from Electronic Health Record Data: Comparison of RECIST, Radiology-Anchored, and Clinician-Anchored Approaches for Abstracting Real-World Progression in Non-Small Cell Lung Cancer | 70 | 14.00 |
|  | CALDWELL SA, 2003, J IMMUNOL | The Fas/Fas ligand pathway is important for optimal tumor regression in a mouse model of CTL adoptive immunotherapy of experimental CMS4 lung metastases | 67 | 3.19 |
|  | NISHINO M, 2016, J IMMUNOTHER CANCER | Immune-related response assessment during PD-1 inhibitor therapy in advanced non-small-cell lung cancer patients | 65 | 8.13 |
| Breast Cancer | PARK SG, 2010, CANCER CELL | The therapeutic effect of anti-HER2/neu antibody depends on both innate and adaptive immunity | 375 | 26.79 |
|  | LE HK, 2009, INT IMMUNOPHARMACOL | Gemcitabine directly inhibits myeloid derived suppressor cells in BALB/c mice bearing 4T1 mammary carcinoma and augments expansion of T cells from tumor-bearing mice | 258 | 17.20 |
|  | CHEN WR, 1997, CANCER LETT | Laser-photosensitizer assisted immunotherapy: a novel modality for cancer treatment | 239 | 8.85 |
|  | MELANI C, 2003, BLOOD | Myeloid cell expansion elicited by the progression of spontaneous mammary carcinomas in c-erbB-2 transgenic BALB/c mice suppresses immune reactivity | 217 | 10.33 |
|  | CHEN WR, 1996, CANCER LETT | Photothermal effects on murine mammary tumors using indocyanine green and an 808-nm diode laser: an in vivo efficacy study | 216 | 7.71 |
| Ovarian Cancer | KERSHAW MH, 2006, CLIN CANCER RES | A phase I study on adoptive immunotherapy using gene-modified T cells for ovarian cancer | 853 | 47.39 |
|  | SHEN J, 2018, NAT MED | ARID1A deficiency promotes mutability and potentiates therapeutic antitumor immunity unleashed by immune checkpoint blockade | 256 | 42.67 |
|  | CANEVARI S, 1995, JNCI-J NATL CANCER INST | Regression of advanced ovarian carcinoma by intraperitoneal treatment with autologous T lymphocytes retargeted by a bispecific monoclonal antibody | 180 | 6.21 |
|  | DEL CARMEN MG, 2005, JNCI-J NATL CANCER INST | Synergism of epidermal growth factor receptor-targeted immunotherapy with photodynamic treatment of ovarian cancer in vivo | 127 | 6.68 |
|  | HERMANSON DL, 2016, STEM CELLS | Induced Pluripotent Stem Cell-Derived Natural Killer Cells for Treatment of Ovarian Cancer | 126 | 15.75 |
